# 5-(2,3-Dichloro­phen­yl)-2-fluoro­pyridine

**DOI:** 10.1107/S1600536812027833

**Published:** 2012-06-23

**Authors:** Fazal Elahi, Muhammad Adeel, M. Nawaz Tahir

**Affiliations:** a Department of Chemistry, Gomal University, Dera Ismail Khan, K.P.K., Pakistan; b University of Sargodha, Department of Physics, Sargodha, Pakistan

## Abstract

In the title compound, C_11_H_6_Cl_2_FN, the dichloro­benzene and the 2-fluoro­pyridine rings are oriented at a dihedral angle of 47.73 (3)°. In the crystal, pairs of C—H⋯N inter­actions link the mol­ecules into dimers with *R*_2_^2^(12) motifs. Mol­ecules are arranged in stacks extending along [100] *via* π–π inter­actions with a centroid–centroid distance of 3.8889 (3) Å.

## Related literature

For related structures, see: Adeel *et al.* (2012 ▶); Elahi, Adeel & Tahir (2012 ▶); Elahi, Adeel, Tahir *et al.* (2012 ▶). For graph-set notation, see: Bernstein *et al.* (1995 ▶).
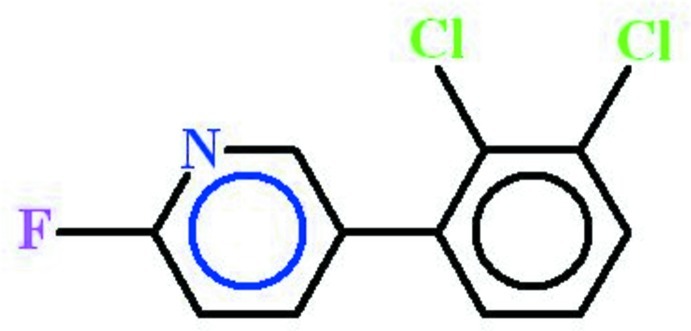


## Experimental

### 

#### Crystal data

C_11_H_6_Cl_2_FN
*M* *_r_* = 242.07
Triclinic, 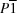

*a* = 3.8889 (3) Å
*b* = 11.1006 (11) Å
*c* = 12.0542 (11) Å
α = 101.526 (5)°
β = 94.930 (4)°
γ = 92.057 (5)°
*V* = 507.24 (8) Å^3^
*Z* = 2
Mo *K*α radiation
μ = 0.61 mm^−1^
*T* = 296 K
0.34 × 0.18 × 0.16 mm

#### Data collection

Bruker Kappa APEXII CCD diffractometer
Absorption correction: multi-scan (*SADABS*; Bruker, 2005 ▶) *T*_min_ = 0.874, *T*_max_ = 0.898
6978 measured reflections
1874 independent reflections
1625 reflections with *I* > 2σ(*I*)
*R*_int_ = 0.023

#### Refinement

*R*[*F* ^2^ > 2σ(*F* ^2^)] = 0.028
*wR*(*F* ^2^) = 0.075
*S* = 1.04
1874 reflections
136 parameters
H-atom parameters constrained
Δρ_max_ = 0.18 e Å^−3^
Δρ_min_ = −0.22 e Å^−3^

### 

Data collection: *APEX2* (Bruker, 2007 ▶); cell refinement: *SAINT* (Bruker, 2007 ▶); data reduction: *SAINT*; program(s) used to solve structure: *SHELXS97* (Sheldrick, 2008 ▶); program(s) used to refine structure: *SHELXL97* (Sheldrick, 2008 ▶); molecular graphics: *ORTEP-3 for Windows* (Farrugia, 1997 ▶) and *PLATON* (Spek, 2009 ▶); software used to prepare material for publication: *WinGX* (Farrugia, 1999 ▶) and *PLATON*.

## Supplementary Material

Crystal structure: contains datablock(s) global, I. DOI: 10.1107/S1600536812027833/gk2505sup1.cif

Structure factors: contains datablock(s) I. DOI: 10.1107/S1600536812027833/gk2505Isup2.hkl

Supplementary material file. DOI: 10.1107/S1600536812027833/gk2505Isup3.cml

Additional supplementary materials:  crystallographic information; 3D view; checkCIF report

## Figures and Tables

**Table 1:** Hydrogen-bond geometry (Å, °)

*D*—H⋯*A*	*D*—H	H⋯*A*	*D*⋯*A*	*D*—H⋯*A*
C6—H6⋯N1^i^	0.93	2.63	3.557 (2)	178

Symmetry code: (i) 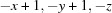.

**Fig. 1.**
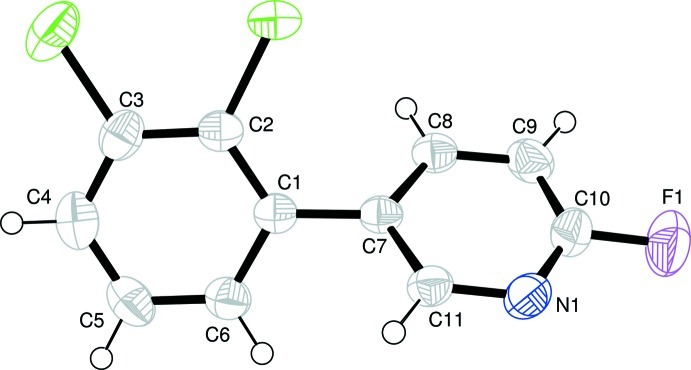
View of the title compound with the atom numbering scheme. Dispalcement ellipsoids are drawn at the 50% probability level. H-atoms are shown by small circles of arbitrary radii.

**Fig. 2.**
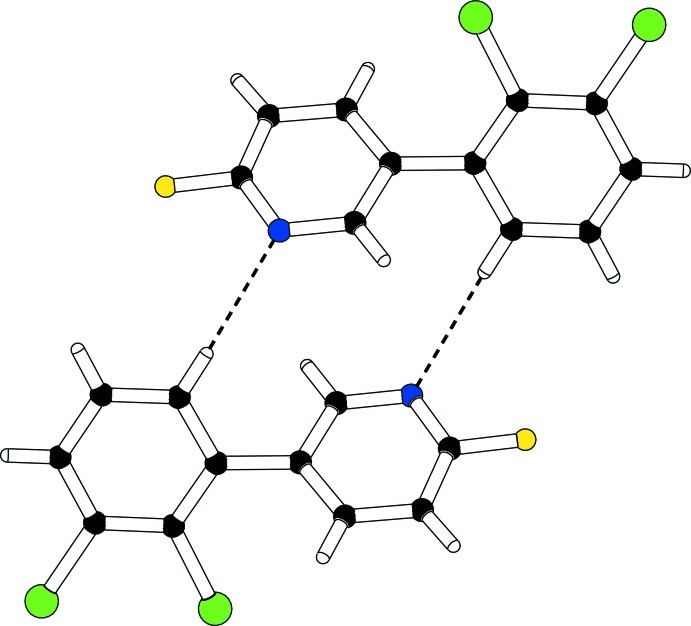
The partial packing (PLATON; Spek, 2009) which shows that molecules form dimers via R22(12) motif.

## supplementary crystallographic information

### Comment

We have reported the crystal structure of 5-(4-chlorophenyl)-2-fluoropyridine (Adeel *et al.*, 2012), 4-(2-fluoropyridin-5-yl)phenol (Elahi, Adeel & Tahir, 2012) and 5-(4-fluorophenyl)-2-fluoropyridine (Elahi, Adeel, Tahir *et al.*, 2012)which have common moiety of 2-fluoropyridine as in (I).

In (I) the dichlorobenzene A (C1–C6/CL1/CL2) and the 2-fluoropyridine B (C7—C11/N1/F1) are planar with r.m.s. deviations of 0.0189 Å and 0.0042 Å, respectively. The dihedral angle between A/B is 47.73 (3)°. The molecules are stabilized in the form of dimers with *R*_2_^2^(12) ring motif (Bernstein *et al.*, 1995) due to hydrogen bonding of C—H···N type between dichlorophenyl and pyridine groups (Table 1, Fig. 2).

### Experimental

To a 6 ml solution of 5-bromo-2-fluoropyridine (0.2 g, 1.136 mmol), 2,3-dichlorophynyl boronic acid (0.260 g, 1.36 mmol) in dioxane and K_3_PO_4_ (0.361 g, 1.5 mmol, in 1 ml H_2_O) was added Pd(PPh_3_)_4_ (1.5 mole %) at 373 K under N_2_ atmosphere. The reaction mixture was refluxed for 8 h. Then 20 ml of distilled water was added. The aqueous layer was extracted three times with ethyl acetate (3 × 15 ml). The organic layer was evaporated in *vacuo* and the crude product was obtained. Colorless needles of (I) were obtained by the recrystalization of crude product in a saturated CHCl_3_/CH_3_OH (m.p. 350-352 K).

### Refinement

The H atoms were positioned geometrically (C–H = 0.93 Å) and refined as riding with *U*_iso_(H) = *xU*_eq_(C), where *x* = 1.2 for aryl H atoms.

### Crystal data

**Table:**

C_11_H_6_Cl_2_FN	*Z* = 2
*M**_r_* = 242.07	*F*(000) = 244
Triclinic, *P*1	*D*_x_ = 1.585 Mg m^−^^3^
Hall symbol: -P 1	Mo *K*α radiation, λ = 0.71073 Å
*a* = 3.8889 (3) Å	Cell parameters from 869 reflections
*b* = 11.1006 (11) Å	θ = 1.7–25.5°
*c* = 12.0542 (11) Å	µ = 0.61 mm^−^^1^
α = 101.526 (5)°	*T* = 296 K
β = 94.930 (4)°	Needle, colorless
γ = 92.057 (5)°	0.34 × 0.18 × 0.16 mm
*V* = 507.24 (8) Å^3^	

### Data collection

**Table:**

Bruker Kappa APEXII CCD diffractometer	1874 independent reflections
Radiation source: fine-focus sealed tube	1625 reflections with *I* > 2σ(*I*)
Graphite monochromator	*R*_int_ = 0.023
Detector resolution: 8.10 pixels mm^-1^	θ_max_ = 25.5°, θ_min_ = 1.7°
ω scans	*h* = −4→4
Absorption correction: multi-scan (*SADABS*; Bruker, 2005)	*k* = −13→13
*T*_min_ = 0.874, *T*_max_ = 0.898	*l* = −14→14
6978 measured reflections	

### Refinement

**Table:**

Refinement on *F*^2^	Primary atom site location: structure-invariant direct methods
Least-squares matrix: full	Secondary atom site location: difference Fourier map
*R*[*F*^2^ > 2σ(*F*^2^)] = 0.028	Hydrogen site location: inferred from neighbouring sites
*wR*(*F*^2^) = 0.075	H-atom parameters constrained
*S* = 1.04	*w* = 1/[σ^2^(*F*_o_^2^) + (0.0353*P*)^2^ + 0.1351*P*] where *P* = (*F*_o_^2^ + 2*F*_c_^2^)/3
1874 reflections	(Δ/σ)_max_ < 0.001
136 parameters	Δρ_max_ = 0.18 e Å^−^^3^
0 restraints	Δρ_min_ = −0.22 e Å^−^^3^

### Special details

**Table:**

Geometry. Bond distances, angles *etc*. have been calculated using the rounded fractional coordinates. All su's are estimated from the variances of the (full) variance-covariance matrix. The cell e.s.d.'s are taken into account in the estimation of distances, angles and torsion angles
Refinement. Refinement of *F*^2^ against ALL reflections. The weighted *R*-factor *wR* and goodness of fit *S* are based on *F*^2^, conventional *R*-factors *R* are based on *F*, with *F* set to zero for negative *F*^2^. The threshold expression of *F*^2^ > σ(*F*^2^) is used only for calculating *R*-factors(gt) *etc*. and is not relevant to the choice of reflections for refinement. *R*-factors based on *F*^2^ are statistically about twice as large as those based on *F*, and *R*- factors based on ALL data will be even larger.

### Fractional atomic coordinates and isotropic or equivalent isotropic displacement parameters (Å^2^)

**Table:**

	*x*	*y*	*z*	*U*_iso_*/*U*_eq_	
Cl1	0.89637 (11)	0.34986 (4)	0.42850 (3)	0.0469 (2)	
Cl2	0.69950 (16)	0.07317 (5)	0.40724 (5)	0.0705 (2)	
F1	0.8508 (4)	0.81300 (10)	0.19925 (12)	0.0787 (5)	
N1	0.8522 (4)	0.61337 (13)	0.12178 (12)	0.0454 (5)	
C1	0.5754 (4)	0.32731 (14)	0.21540 (13)	0.0333 (5)	
C2	0.6680 (4)	0.26882 (14)	0.30479 (13)	0.0355 (5)	
C3	0.5869 (4)	0.14425 (15)	0.29533 (15)	0.0441 (6)	
C4	0.4176 (5)	0.07576 (17)	0.19693 (18)	0.0535 (6)	
C5	0.3322 (5)	0.13175 (16)	0.10746 (17)	0.0518 (6)	
C6	0.4073 (4)	0.25512 (15)	0.11605 (14)	0.0421 (5)	
C7	0.6503 (4)	0.46004 (14)	0.21958 (12)	0.0329 (5)	
C8	0.5751 (4)	0.55329 (15)	0.30850 (13)	0.0393 (5)	
C9	0.6419 (5)	0.67424 (16)	0.30366 (15)	0.0469 (6)	
C10	0.7801 (5)	0.69556 (15)	0.20828 (16)	0.0475 (6)	
C11	0.7863 (4)	0.49632 (15)	0.12855 (13)	0.0383 (5)	
H4	0.36156	−0.00758	0.19106	0.0642*	
H5	0.22187	0.08546	0.04010	0.0621*	
H6	0.34500	0.29133	0.05456	0.0505*	
H8	0.47957	0.53360	0.37117	0.0472*	
H9	0.59579	0.73832	0.36209	0.0562*	
H11	0.83512	0.43519	0.06797	0.0460*	

### Atomic displacement parameters (Å^2^)

**Table:**

	*U*^11^	*U*^22^	*U*^33^	*U*^12^	*U*^13^	*U*^23^
Cl1	0.0512 (3)	0.0576 (3)	0.0312 (2)	0.0030 (2)	−0.0007 (2)	0.0092 (2)
Cl2	0.0884 (4)	0.0636 (3)	0.0719 (4)	0.0107 (3)	0.0127 (3)	0.0404 (3)
F1	0.1164 (11)	0.0382 (6)	0.0853 (9)	−0.0001 (6)	0.0163 (8)	0.0196 (6)
N1	0.0540 (9)	0.0448 (8)	0.0408 (8)	0.0054 (7)	0.0071 (7)	0.0155 (6)
C1	0.0300 (8)	0.0385 (8)	0.0319 (8)	0.0047 (6)	0.0071 (6)	0.0060 (6)
C2	0.0325 (8)	0.0425 (9)	0.0322 (8)	0.0039 (6)	0.0076 (6)	0.0068 (6)
C3	0.0422 (10)	0.0445 (9)	0.0512 (10)	0.0069 (7)	0.0130 (8)	0.0185 (8)
C4	0.0530 (11)	0.0382 (9)	0.0687 (13)	−0.0011 (8)	0.0091 (9)	0.0089 (9)
C5	0.0497 (11)	0.0452 (10)	0.0524 (11)	−0.0033 (8)	−0.0035 (8)	−0.0045 (8)
C6	0.0422 (9)	0.0444 (9)	0.0383 (9)	0.0049 (7)	0.0011 (7)	0.0058 (7)
C7	0.0309 (8)	0.0383 (8)	0.0294 (8)	0.0051 (6)	0.0019 (6)	0.0064 (6)
C8	0.0409 (9)	0.0446 (9)	0.0323 (8)	0.0049 (7)	0.0062 (7)	0.0062 (7)
C9	0.0555 (11)	0.0409 (9)	0.0407 (10)	0.0093 (8)	0.0031 (8)	−0.0007 (7)
C10	0.0558 (11)	0.0360 (9)	0.0519 (10)	0.0019 (8)	−0.0005 (8)	0.0141 (8)
C11	0.0428 (9)	0.0407 (9)	0.0323 (8)	0.0073 (7)	0.0051 (7)	0.0079 (7)

### Geometric parameters (Å, º)

**Table:**

Cl1—C2	1.7323 (16)	C5—C6	1.372 (2)
Cl2—C3	1.7271 (18)	C7—C8	1.392 (2)
F1—C10	1.350 (2)	C7—C11	1.381 (2)
N1—C10	1.299 (2)	C8—C9	1.372 (2)
N1—C11	1.335 (2)	C9—C10	1.368 (3)
C1—C2	1.394 (2)	C4—H4	0.9300
C1—C6	1.396 (2)	C5—H5	0.9300
C1—C7	1.482 (2)	C6—H6	0.9300
C2—C3	1.387 (2)	C8—H8	0.9300
C3—C4	1.374 (3)	C9—H9	0.9300
C4—C5	1.371 (3)	C11—H11	0.9300
			
C10—N1—C11	115.72 (15)	C8—C9—C10	116.44 (16)
C2—C1—C6	117.42 (15)	F1—C10—N1	114.38 (16)
C2—C1—C7	123.81 (14)	F1—C10—C9	118.82 (16)
C6—C1—C7	118.76 (14)	N1—C10—C9	126.79 (17)
Cl1—C2—C1	120.44 (12)	N1—C11—C7	124.31 (15)
Cl1—C2—C3	118.82 (12)	C3—C4—H4	120.00
C1—C2—C3	120.72 (15)	C5—C4—H4	120.00
Cl2—C3—C2	120.27 (13)	C4—C5—H5	120.00
Cl2—C3—C4	119.16 (14)	C6—C5—H5	120.00
C2—C3—C4	120.57 (16)	C1—C6—H6	119.00
C3—C4—C5	119.21 (18)	C5—C6—H6	119.00
C4—C5—C6	120.85 (18)	C7—C8—H8	120.00
C1—C6—C5	121.20 (16)	C9—C8—H8	120.00
C1—C7—C8	123.79 (14)	C8—C9—H9	122.00
C1—C7—C11	119.39 (14)	C10—C9—H9	122.00
C8—C7—C11	116.74 (15)	N1—C11—H11	118.00
C7—C8—C9	119.99 (15)	C7—C11—H11	118.00
			
C11—N1—C10—F1	179.73 (16)	Cl1—C2—C3—C4	−177.52 (14)
C11—N1—C10—C9	−0.7 (3)	C1—C2—C3—Cl2	−179.38 (12)
C10—N1—C11—C7	0.0 (2)	C1—C2—C3—C4	0.9 (2)
C6—C1—C2—Cl1	176.78 (12)	Cl2—C3—C4—C5	−179.13 (15)
C6—C1—C2—C3	−1.6 (2)	C2—C3—C4—C5	0.6 (3)
C7—C1—C2—Cl1	−2.2 (2)	C3—C4—C5—C6	−1.4 (3)
C7—C1—C2—C3	179.45 (15)	C4—C5—C6—C1	0.6 (3)
C2—C1—C6—C5	0.9 (2)	C1—C7—C8—C9	−177.67 (16)
C7—C1—C6—C5	179.88 (16)	C11—C7—C8—C9	−1.0 (2)
C2—C1—C7—C8	−49.6 (2)	C1—C7—C11—N1	177.60 (15)
C2—C1—C7—C11	133.83 (17)	C8—C7—C11—N1	0.8 (2)
C6—C1—C7—C8	131.43 (17)	C7—C8—C9—C10	0.5 (3)
C6—C1—C7—C11	−45.1 (2)	C8—C9—C10—F1	−179.97 (19)
Cl1—C2—C3—Cl2	2.22 (19)	C8—C9—C10—N1	0.5 (3)

### Hydrogen-bond geometry (Å, º)

**Table:**

*D*—H···*A*	*D*—H	H···*A*	*D*···*A*	*D*—H···*A*
C6—H6···N1^i^	0.93	2.63	3.557 (2)	178

Symmetry code: (i) −*x*+1, −*y*+1, −*z*.
